# Evaluation of biological markers for the risk assessment of carbon black in epidemiological studies

**DOI:** 10.3389/fpubh.2024.1367797

**Published:** 2024-04-16

**Authors:** Mei Yong, Robert J. McCunney

**Affiliations:** ^1^MY EpiConsulting, Duesseldorf, Germany; ^2^Brigham and Women’s Hospital, Pulmonary Division, Harvard Medical School, Boston, MA, United States

**Keywords:** epidemiological studies, nanomaterials, carbon black, biological markers, inflammatory markers, cross-sectional studies, causality

## Abstract

**Background/objectives:**

Engineered nanomaterials (ENMs) have been suggested as being capable of promoting inflammation, a key component in the pathways associated with carcinogenesis, cardiovascular disease, and other conditions. As a result, the risk assessment of biological markers as early-stage indicators has the potential to improve translation from experimental toxicologic findings to identifying evidence in human studies. The study aims to review the possible early biological changes in workers exposed to carbon black (CB), followed by an evidentiary quality evaluation to determine the predictive value of the biological markers.

**Methods:**

We conducted a literature search to identify epidemiological studies that assessed biological markers that were involved in the inflammatory process at early stages among workers with exposure to CB. We reviewed the studies with specific reference to the study design, statistical analyses, findings, and limitations.

**Results:**

We identified five Chinese studies that investigated the potential impact of exposure to CB on inflammatory markers, bronchial wall thickening, genomic instability, and lung function impairment in CB production workers. Of the five Chinese studies, four were cross-sectional; another study reported results at two-time points over six years of follow-up. The authors of all five studies concluded positive relationships between exposure and the inflammatory cytokine profiles. The weak to very weak correlations between biomarkers and early-stage endpoints were reported.

**Conclusion:**

Most inflammatory markers failed to satisfy the proposed evidentiary quality criteria. The significance of the results of the reviewed studies is limited by the cross-sectional study design, inconsistency in results, uncertain clinical relevance, and high occupational exposures. Based on this review, the risk assessment relying on inflammatory markers does not seem appropriate at this time. Nevertheless, the novel research warrants further exploration in assessing exposure to ENMs and corresponding potential health risks in occupational settings.

## Introduction

With the increasing production and use of engineered nanomaterials (ENMs), it is crucial to understand and evaluate ENMs regarding their possible health impacts. Carbon black (CB) is a powdered form of elemental carbon (EC) with a primary particle size of 10–300 nanometers (nm). CB particulates are usually highly agglomerated, with 10–1,000 particle aggregates per agglomerate having aerodynamic diameters of up to 200–400 nm. Based on the definition of the European Commission in 2011 that 50% of the primary particles (constituent of the aggregate) measured in the number size distribution have a diameter that is less than or greater than 100 nm, commercially available CB grades fall into one of the two categories: nanoforms of CB or non-nanoforms of CB, with the vast majority of CB grades falling into the nanoform category (1). In occupational inhalation exposure scenarios, CB particles satisfying the nano definition form agglomerates. After deposition in the lungs, these particles do not translocate to remote organs from the lungs (2).

In addition to its structure and fine particulate nature, CB gained regulatory attention since the European Commission is currently assessing substances categorized as poorly soluble particulates with low toxicity (PSLT), including titanium dioxide (TiO2) and CB, regarding their potential carcinogenic effect in humans through inhalation. PSLTs are considered chemically inert and are without known specific or inherent toxicity, i.e., a lack of biochemical reaction between the molecules at their surface or dissolved from their surface and the embedding lung fluid (3, 4).

Considering the conceptual mechanism, inflammation is a potential key factor in any potential carcinogenic pathway associated with CB. Inflammatory responses are a dynamic process that is a normal response to prevent disease, however may instead cause disease, if it is inappropriate and/or uncontrolled. Excessive exposure to CB in rodents causes chronic inflammation that may result in impaired particle clearance and increased particle retention, a phenomenon described as “lung overload” in the alveolar region of the lung, and consequently induces tumors in rats, but not in mice and hamsters. The research group of Mauderly et al. and Nikula et al. found an increase in lung tumors in all the exposed groups of rats to elevated concentrations of diesel exhaust and CB particles (5, 6). Heinrich et al. conducted similar studies, in which exposure to CB was used as an inert control particulates in a study of diesel engine exhaust particulates (7) or to tar pitch condensation aerosols (8). In both studies, long-term exposures to CB particles produced lung tumors in the rats. Contrary to the rats study meta-analyses of large occupational cohort studies of CB production workers do not indicate elevated mortality for lung cancer (9) or cardiovascular diseases (10).

The results from animal inhalation studies with PSLTs like CB are limited in their appropriateness for direct extrapolation to human risk because of differences in deposition and dosimetry between rats and humans, among others. Translational toxicology might not be the appropriate method to interpret all the inter-species differences in terms of mode of action (MoA) and adverse outcome pathways (AOPs). The epidemiological investigation of early outcomes at the physiological level will provide more insight into a potential AOP in humans, if present.

### Biological plausibility of inflammatory markers to predict lung cancer

The European Center for Ecotoxicology and Toxicology of Chemicals (ECETOCs) proposed that in particle-overload exposed rats, continuous exposures promote enhanced transfer of particles to lymph nodes, the accumulation of particles in the lung, increases in lung weight, the alveolar macrophage accumulation, pulmonary inflammation, the alveolar epithelial hyperplasia (proliferation), metaplasia, fibrosis, and eventually leads to cancer (ECETOC, 2013). These findings are consistent with scientific evidence that supports a threshold model for PSLT particles causing adverse respiratory tract effects in humans (11, 12).

Conceptual mechanisms of inhalation-mediated cardiovascular toxicity include the activation of pro-inflammatory pathways, i.e., the involvement in the upregulation of inflammatory reactions, and/or oxidative stress, alterations in autonomic balance, and potentially direct actions upon the vasculature of particle constituents capable of reaching the systemic circulation (13). The potential relationship between exposure to airborne particulates and cardiovascular diseases has been investigated in environmental epidemiological studies and some occupational cohorts. The American Heart Association (AHA) published a position paper on particulate matter (PM) and heart disease and considered PM_2.5_ to be a trigger of cardiovascular morbidity and mortality (14). The European Society of Cardiology has drawn similar conclusions (15). Moreover, AHA indicated that the ultrafine particulates from air pollution elicit numerous biological responses, including systemic inflammation, which may further augment future cardiovascular risk over long-term exposure (14).

### The value of biomarkers in risk assessment

While biomarkers, in particular inflammatory markers, are still novel in the risk assessment of chemical substances, their conceptual benefits have been thoroughly addressed in the drug discovery, development, and approval process.

Along with the contentious application of biomarkers to support regulatory decisions, one of the major challenges in biomarker research is to reliably identify a suitable biomarker for guiding clinical and regulatory decisions. The concepts of biomarker qualification and validation processes have been developed by the US Food and Drug Administration – National Institutes of Health (FDANIH).

Biomarkers, EndpointS, and other Tools (BEST) resource was developed in 2016 by the FDANIH to clarify and harmonize terminology and thus promote research, development, and testing of novel methodologies in particular biomarkers (16). According to BEST, biomarkers are defined as “A defined characteristic that is measured as an indicator of normal biological processes, pathogenic processes, or responses to an exposure or intervention, including therapeutic interventions” (17).

The BEST resource defines a surrogate marker as a “laboratory measurement or physical sign that is used in therapeutic trials as a substitute for a clinically meaningful endpoint” (BEST, 2016). The majority of known biomarkers lack sufficient data to prove the direct relation between the level of a biomarker and the clinical outcome; that is, the change in the biomarker does not necessarily explain the change in the clinical outcome (18).

Analogous to drug development, the use of suitable biomarkers in risk assessment can contribute to understanding mechanisms, detecting early biological changes, identifying sensitive workers, monitoring and predicting toxicity issues, and finally enhancing the safe use of potentially hazardous materials significantly. The concept and the validation process can be adopted in the risk assessment of chemical substances.

To date, limited human data can be found on the biological changes at the early stages induced by ENMs. A former review (19) first assessed the value of biomarkers based on epidemiological studies, including several ENMs, such as nanosilver, carbon nanotubes, silica dioxide, TiO2, and nanoresin. The authors reviewed biological markers used in various studies, including, cardiovascular, lung fibrosis, pulmonary inflammation, systemic inflammatory markers, nucleic acids, lipid and protein oxidative stress markers, antioxidant enzyme activity, and genotoxicity markers. However, CB was excluded since the authors were uncertain about the categorization of CB as an ENM (19). To address this gap in the literature, we aimed to identify and review the recent studies focusing on biological changes among CB-exposed workers populations.

The primary objective of this review is to critically evaluate the possible relationship between occupational exposure to CB, inflammatory responses, and possible clinical outcomes. For each possible association, we addressed three key questions: (1) Are the findings clinically relevant? (2) Are these findings consistent? (3) Are there reliable links to support the conceptual AOP?

## Materials and methods

This review was conducted according to the principles of the Preferred Reporting Items for Systematic Reviews and Meta-Analysis (PRISMA) checklist (20). We searched the scientific literature to identify epidemiological studies of workers exposed to CB, in which inflammatory responses were assessed, either systemic or specific, in target organs. Keywords used in our search included “carbon black,” “health effect,” “biomarkers,” and “epidemiology” to identify related publications from PubMed and Medline through December 2023. Animal and *in vitro* studies were excluded from this review. We focused on the methodological quality assessment of each study identified and integrated the evidence across the studied mechanistic pathways.

We adopted the evidentiary criteria of biomarkers used in drug approval processes, which were developed based on *“Biomarker Qualification Workshop: Framework for Defining Evidentiary Criteria”* organized by the FNIH Biomarkers Consortium/FDA in 2016 (BEST, 2016). The purpose of the workshop was to enhance the clarity, predictivity, and harmonization of biomarker qualification with a standard framework.

According to the defined evidentiary criteria, a number of aspects of the FNIH/FDA Workshop is shown in Table 1 Leptak et al. (21).

**Table 1:** Evidentiary criteria of biomarkers according to the BEST resource.*

I. Characterization of the relationship between the biomarker and clinical outcome.
In the risk assessment of PSLTs, the purpose is to identify biomarkers to diagnose the biological changes at early stages or to predict the clinical outcomes at advanced stages.
II. Biological rationale for the use of biomarker (if known)
*In vivo* and/or *in vitro* findings that are usually given before a hypothesis is postulated in epidemiological research.
III. Type of data and study design
According to the study design, data can be categorized into descriptive, cross-sectional, prospective, or retrospective studies.
IV. Independent data sets for qualification
A sufficient amount of independent data sets from different production conditions, exposure scenarios provide consistent findings.
V. A comparison to the current standard. A thorough evaluation of the risk and benefit relationship will be necessary.
VI. Assay performance
Validated tests with high accuracy are generally expected.
VII. Statistical methods
A careful evaluation of statistical approaches is critical because some assumptions/claims may be unfounded if based on inappropriate statistical analyses.

* Adapted from this reference.

## Results

Among the 112 studies identified by our search terms, 5 Chinese studies met the search criteria. These studies investigated the potential impact of exposure to CB on inflammatory markers, bronchial wall thickening, genomic instability, and lung function impairment in CB production workers.

Exposed populations were recruited from acetylene black production facilities (22–26).

For the CB packers from an acetylene black production site, the median exposure duration was 9.3 years, ranging from 1.3 to 31 years (23). The mean concentration of CB measured by personal air samples was 14.9 mg/m^3^ (23, 24).

The CB exposure of the packers from the manufacturing plant was assessed by PM_2.5_, PM_2.5_-related EC, organic carbon (OC), and total carbon (TC). The results of air samplings were reported at two-time points. Dai et al. (26) reported measurements from 2012, which yielded an average of 800 μg/m^3^ for PM_2.5_ and an average of 657.0 μg/m^3^ for PM_2.5_-related EC. Cheng et al. (25) reported the measurements from the visit in 2018, which yielded a geometric mean of 637.4 μg/m^3^ for total PM_2.5_, and 364.6 μg/m^3^ for PM_2.5_. The ratio of PM_2.5_-related EC to TC is 92.5% in two air samples collected from CB bagging areas.

Table 2 provides an overview of the five reviewed studies. Later, we will discuss the evidentiary quality of the studies.

**Table 2:** Epidemiological studies of inflammatory responses in workers with exposure to carbon black.

Studies	Workplace/operations	Study design	Exposed/non-exposed workers	Biomarkers	Clinical findings
Zhang et al. (24)	CB baggers from an acetylene black facility	Cross-sectional	Male packers exposed to acetylene black (*N* = 81); non-exposed male workers (*N* = 104)	Pro-inflammatory cytokines levels: Interleukin(IL)-1ß, IL-6, MIP-1ß, and TNF-alpha	Increased pro-inflammatory cytokines levels among the exposed. No changes found in chest X-ray images; Significant, though no pathological reduction of lung function parameters.
Dai et al. (26)	Packing in an acetylene black facility	Cross-sectional	Male packers exposed to pure CB (*N* = 106); non-exposed controls from a water plant in the same region, frequency-matched by age and smoking status (*N* = 112)	Peripheral blood cell counts and lymphocyte subsets; IL-1ß, IL-6, and MIP-1ß	In total, 30.8% of increased peripheral blood eosinophil count, though in the normal range. No significant difference in other cell counts. Positive correlations between cell counts and the levels of cytokines in the serum of exposed workers with correlation coefficient ranging from 0.20 to 0.27
Yang et al. (23)	CB baggers from an acetylene black facility	Cross-sectional	*N* = 99, male packers; *N* = 115, healthy male controls	Markers for peripheral cell damage and pulmonary blood barrier permeability: clara cell secretory protein (CC16) and surfactant-associated protein (SP-A)	A 20% decrease in CC16 and a 15% increase in SP-A among the CB packers. No consistent trends concerning CC16 and SP-A and lung functions, in the subgroups according to the exposure status
Cheng et al. (25)	Packing in an acetylene black facility	Cross-sectional, longitudinal	At baseline in 2012: 85 CBPs vs. 106 Non-CBPs; 2018 FU visit: 53 CBPs vs. 55 non-CBPs	Carbon content in airway macrophage (CCAM); proportion of cytoplasm area occupied by carbon particles (PCOC); IL-1ß, IL-6, IL-8, MIP-1ß, CRP; and TNF-alpha; cytokinesis-block micronucleus (CBMN)	PCOC level: CBP-C > CBP-F > Non-CBP; Positive linear relationship between PCOC and CBMN endpoints
Cao et al. (22)	CB packers from an acetylene black facility	Cross-sectional	*N* = 58 current CB packers (CBP); 95 non-CBP	Carbon content in airway macrophage (CCAM); small airway wall thickness measured by wall area percent (WA%)	Significant, but very weak to weak correlation between CCAM and WA% (0.20 to 0.26), also negligible correlations between WA% and lung function parameters (from −0.07 to −0.26)

In the first study (24), the authors evaluated the exposure to CB and the possible alteration of lung function and inflammatory responses. An exposed group of 81 CB packers was compared with 104 controls. The average age, body mass index (BMI), smoking and drinking habits, employment duration, and shift work status were not statistically significantly different. Zhang et al. reported a statistically significant increase in pro-inflammatory cytokines levels (IL-1ß, IL-6, IL-8, MIP-1ß, and TNF-alpha) and reduced lung function parameters (FEV_1_%, FEV_1_/FVC, MMF%, and PEF, FVC marginally non-significant) among the CB-exposed workers. The results of lung function parameters, however, were within the normal range among the exposed workers and thus of uncertain clinical significance.

In a study at another CB manufacturing plant, Dai et al. aimed to address a similar question. Dai et al. (26) evaluated relationships between occupational exposure to CB and blood cell counts, lymphocyte subsets, and the levels of cytokines in the serum. The authors recruited 106 packers with exposure to CB, compared with 112 unexposed male workers whose frequency matched by age and smoking status at a water plant in the same region. The distributions of age, BMI, and history of smoking and alcohol consumption were balanced. Eosinophil counts increased among the exposed group, although the difference did not reach the statistical significance level (*p* = 0.072) after adjustment for age, BMI, smoking status, and alcohol use. Noticeably, the relationship between serum cytokines and CB exposure was not reported.

Concerning the relationship between blood cell counts and serum cytokine levels, correlation analyses were used. Pearson’s correlation coefficients between blood cell counts and the levels of cytokines ranged from 0.2 to 0.27, with statistical significance. However, the magnitude of the correlation coefficients indicated very weak correlations. Thus, a link between white blood cell counts and the levels of pro-inflammatory cytokines was not supported with confidence.

In a follow-up study based on the same study population as Yang et al. (23) and Zhang et al. (24) focused on the potential impact of CB exposure on peripheral cell damage and pulmonary blood barrier permeability by means of Clara cell secretory protein (CC16) and surfactant-associated protein (SP-A) levels in relation to lung function parameters. However, the numbers of the study population showed a minor discrepancy, consisting of 99 CB-exposed and 115 controls. The authors reported a 20% decrease in CC16 and a 15% increase in SP-A among the CB packers, along with reduced lung function parameters (FEV_1_/FVC, MMF%, PEF25%, and PEF75%, *p* < 0.05).

To explore the relationship with lung function, the authors categorized the serum CC16 and SP-A levels into tertiles. Lung function parameters as a dependent variable were then adjusted for age, BMI, smoking status, and alcohol consumption. In all subjects combined, positive associations were found between CC16 and all the lung function parameters, whereas negative associations between SP-A and lung function parameters.

The trends could not be recognized after dividing the subjects according to the exposure status, neither in the CB-exposed group nor in the control group. Thus, there is no suggestion that exposure to CB would modify the link between CC16 and SP-A and lung functions.

In a further study, Cheng et al. (25) studied the potential link between CB exposure and genomic instability in peripheral lymphocytes with the use of cytokinesis-block micronucleus (CBMN) endpoints, since the CBMN assay was thought to detect injuries that survive at least one metonic cycle and reflect unrepaired fixed DNA damage.

The investigators measured carbon content in airway macrophages (CCAMs) in sputum as a proxy for determining chronic exposure to CB aerosol and PM (PM_2.5,_ PM_2.5_-related EC) in air pollution. The proportion of cytoplasmic area occupied by carbon particles (PCOC) is a quantification of the carbon content in each macrophage. The investigators reported an apparent gradient of PCOC in current CB packers compared to former CB packers and compared to controls. CBMN was used to quantify the unrepaired DNA damage after injuries that resulted from exposure. The measurements of CBMN endpoints were reported both for the 2012 visit and replicated for the 2018 follow-up. The authors described the 232 study subjects as cohort members but analyzed the data in accordance with the cross-sectional study design, with no consideration of change over time from a longitudinal perspective. At both time points, significantly increased levels of CBMN endpoints were reported. A marked increase was observed among current CB packers compared to former CB packers and compared to non-exposed. A positive linear relationship between PCOC and CBMN was reported, after adjustment for age, BMI, current smoking and drinking status, and pack years. Furthermore, the investigators used mediation analyses to evaluate the mediating effect of circulatory inflammation on the relationship between CB exposure and CBMN endpoints.

In the following study, Cao et al. (22) studied CCAM in sputum in relationship with bronchial wall thickness measured by wall area percent (WA%) of the sixth and ninth generations of airways, which represents a measure for airway remodeling. Elevated levels of CCAM in sputum were found among CB packers, both for ever smokers and never smokers. Significantly increased WA% was found in CB packers vs. non-CB packers for both generations of airways. Interestingly, cigarette smoking status and pack years showed no effect on any of these measurements. Using correlation analyses, they found weak to very weak correlations between the CCAM and wall thickness (WA%), with significant correlation coefficients ranging from 0.20 to 0.26 (*cf.* Figure 3 from (22)). Furthermore, they reported very weak correlations between WA% and pulmonary function parameters, with correlation coefficients ranging from −0.07 (*p* = 0.38) to −0.26 (*p* = 0.001) (*cf.* Figure 4 from (22)). The weak to very weak correlations between CCAM and WA% could not support a certain relationship between CB exposure and wall thickness. Similarly, the correlation between WA% and pulmonary function parameters was weak to very weak.

### Evaluation of evidentiary quality of the studies

Table 3 shows the evaluation of the individual studies according to the BEST quality criteria proposed by the FNIH/FDA.

I. In consideration of the characterization of the relationship between the biomarker and clinical outcome, the inflammatory markers should predict the clinical outcome at advanced stages of biological processes. Zhang et al. (24) and Dai et al. (26) made comparisons between the CB-exposed workers and controls. Prediction was not the subject of the studies.
II. The biological rationale for the postulated hypotheses was given based on the findings from in vivo or in vitro studies.
III. In light of the data type and study design, four studies used a cross-sectional design; one study reported results from two visits, in 2012 and 2018. Thus, an intra-individual comparison in a longitudinal manner was feasible. However, an intra-individual comparison was neither performed nor reported by the authors. We tentatively compared the changes between the groups in Table 4, implying that the worsening effect among the CBPs might actually be slower than the controls over the next 6 years.
IV. The data included in this review were highly dependent. Exposed populations were recruited from two workplaces. Independent data from other populations and exposure scenarios would enhance the statistical power and consistency of the findings.
V. The current standard definition of an adverse event relied on clinically manifested outcomes. Using biological markers has the potential of added value. A thorough consideration of the risk–benefit relationship would be necessary, both for the researchers and for the regulators. To address the accuracy of a biological marker, both sensitivity and specificity need to be addressed, to minimize false-positive or false-negative results. In this series of studies, only sensitivity was analyzed.
VI. The author believed in using the validated test assay.
VII. Correlation analysis is the most commonly used statistical approach to quantifying the strength of relationships. Mediation analysis is used to test and estimate the mediation effects of the studied biological markers on transmission from exposure to its effects. The conditions for performing a mediation analysis are explained in the discussion.

**Table 3:** The evaluation of the individual studies according to the evidentiary quality criteria.

Quality criteria		Zhang et al. (24)	Dai et al. (26)	Yang et al. (23)	Cheng et al. (25)	Cao et al. (22)
I	Characterization	No prediction	No prediction	Explore the link to AOP	Explore the link to AOP	Explore the link to AOP
II	Biological rationale	Given	Given	Given	Given	Given
III	Study design	Cross-sectional	Cross-sectional	Cross-sectional	Cross-sectional design, with repeated measurements	Cross-sectional
IV	Independent data, consistent findings	Limited data	Limited data	No consistent trends	Inconsistent findings	Weak to very weak correlations
V	Comparison to standard	Cytokine profile	Peripheral white cell counts	Epithelial cell integrity	Genomic instability	Bronchial wall thickness
VI	Assay performance	Valid	Valid	Valid	Valid	Valid
VII	Statistical methods	Multivariate analyses for serum cytokines with adjustment for confounders; Univariate analyses for lung function parameters	Correlation analyses yielded weak to very weak correlations	Categorized analyses; subgroup analyses; With adjustment for confoundsers	Correlation analyses; Mediation analysis	Correlation analyses; Mediation analysis

**Table 4:** Comparisons of within-group changes of cytokinesis-block micronucleus (CBMN) endpoints [abstracted from Cheng et al. (25), Table 1].

Endpoints	Controls			Current carbon black packers		
	2012	2018	Ratio 2018/2012	2012	2018	Ratio 2018/2012
MN (‰)	4.53	6.11	1.35	8.19	7.16	0.87
NPB (‰)	0.29	0.46	1.59	0.51	0.74	1.45
BUD (‰)	0.89	4.38	4.92	2.62	6.26	2.39
CBMN (‰)	5.66	10.95	1.93	11.29	14.16	1.25
NDI	1.82	1.40	0.77	1.79	1.44	0.80

MN, micronucleus; NPB, nucleoplasmic bridge; BUD, nuclear bud; NDI, nuclear division index; CBMN, cytokinesis-block micronucleus.

## Discussion

The present paper reviewed human studies that investigated biological changes in the early phase after occupational exposure to CB, with the purpose of evaluating the biomarkers and their predictive values for clinical outcomes. Five epidemiologic studies from two Chinese research groups provided the first wave of evidence. The study subjects of three studies (22, 24, 25) were male packers in an acetylene black production facility compared to non-exposed male workers. The investigators found statistically significant increases in pro-inflammatory cytokine levels and reduced but not abnormal lung function parameters (24). Yang et al. reported a 20% decrease in CC16 and a 15% increase in SP-A, markers for peripheral cell damage, and reduced lung function among the exposed workers. Positive relationships were found between CC16 and lung function parameters; similarly, negative relationships were found between SP-A and lung function parameters among the combined study subjects. In stratifying the subjects according to exposure status, no trends were observed. The modifying effect of exposure to CB could not be concluded. In the following study, weak to very weak correlations were found between CCAM and bronchial wall thickness (WA%), and WA% with lung function parameters as well (22).

Another group of researchers recruited packers exposed to acetylene black compared with non-exposed packers from a water plant in the same region, with frequency matching by age and smoking status. In Dai et al.’s study (26), no peripheral blood cell count except eosinophils were found to be increased but not in an abnormal range. The correlations between blood cells and pro-inflammatory cytokines that are involved in the upregulation of inflammatory reactions were weak to very weak, despite statistical significance. Furthermore, the authors found noticeable outcomes regarding increased CBMN endpoints, both in 2011 and 2018 visits (25).

### Inconsistency across the reviewed studies

Considering the conceptual AOP after exposure to CB, we found inconsistent and contradictory findings regarding associations between (i) white blood cells and pro-inflammatory cytokines levels, (ii) CCAM and WA%, and (iii) WA% and lung function parameters. The weak to very weak correlations indicated that the clinical relevance of the results is still uncertain, despite some statistical significance.

The inconsistent findings do not support a full understanding of the complex mechanisms for ENM-induced health effects. The investigators of the reviewed papers postulated that inflammation was an adverse event that may either initiate or promote a carcinogenic pathway. In Poland et al.’s study (27), the authors raised the concern of whether pulmonary inflammation is a valid predictor of particle-induced lung pathology, based on case analyses of two particulates. Considering the technological advancement and increased sensitivity of biomarkers, the authors pointed out the challenges in the risk assessment relying on biomarkers: (i) to distinguish an “effect” from an “adverse effect” and (ii) a valid predictor must be sensitive and specific as well.

To evaluate biological markers or pre-clinical endpoints in contrast to manifested clinical outcomes, quality criteria for evaluations are needed for clinical and regulatory decisions.

### Evaluation of evidentiary quality of the studies

In all reviewed studies, exposure was dichotomized into CB exposed and non-exposed in all statistical analyses. Quantitative assessment of exposure measures, for example, mass or surface area of the nanoparticles, was not the subject of the analyses; therefore, the reviewed studies could not explain the relationship between the dose and inflammatory responses and lung function outcomes in CB-exposed workers.

This review of the Chinese cross-sectional studies identified showed elevated pro-inflammatory cytokines among workers with considerably high exposure to CB. The mean concentration of 14.9 mg/m^3^ measured by personal air samples was up to 4-fold greater than the American Conference of Governmental Industrial Hygienists (ACGIH) threshold limit value (TLV) value for CB of 3 mg/m^3^ (inhalable fraction) or worldwide CB OELs which generally range from 2-4 (3 mg/m^3^) as the inhalable fraction (1).

Furthermore, it is essential to distinguish statistical significance from clinical relevance. As shown in Table 2, Zhang et al. (24) reported reduced, but not abnormal (i.e., values below the lower limit of normal), lung function parameters. Similarly, elevated eosinophil counts, but not in the pathological range (28), were reported in Dai et al.’s study Dai et al. (26); significant, but weak correlations were observed between CCAM and WA% (correlation coefficient ranging from 0.20 to 0.26). The very weak to weak correlations were reported between WA% and lung function parameters (correlation coefficient ranging from −0.07 to −0.26) in Cao et al.’s study (22). Pearson’s correlation coefficient is a measure of the correlation between two sets of data, ranging from −1 to 1 (29). Generally, a correlation coefficient of less than 0.7 is not considered strong (30). Thus, a statistically significant correlation but weak in magnitude would not imply certainty of clinical relevance.

In the studies of Cao et al. (22) and Cheng et al. (25), the authors used mediation analyses to explain and estimate the underlying mechanism of the inflammatory markers on the pathway of exposure to CB and biological outcomes. To estimate and test the mediation effects of the biomarkers, the validity of the following three steps is required: (i) exposure–mediator, (ii) mediator–outcome, and (iii) exposure–outcome (31). In Cheng et al.’s study (25), 6 out of 15 tests noted a statistically significant mediation effect. A clear pattern, however, was not presented. In Cao et al.’s study (22), the authors found statistically significant—but weak—associations between airway wall thickening (WA%) and spirometry parameters, while none of the associations between CB exposure and spirometry parameters were statistically significant. It is noted that the association of the main pathway, here CB exposure to the outcome, is not provided; thus, the required assumption (iii) of the mediation analysis is therefore not fulfilled. Hence, the validity of the statistical handling and the resulting findings to estimate the mediation effects seem doubtful.

Finally, the major limitation of the studies is the cross-sectional study design, which inherently cannot conclude causality. Only one study, Cheng et al. (25), reported results from two subsequent visits. Comparing the ratios of the respective biomarkers from 2018 to 2012 in Table 4, our tentative analysis considering within-group changes in a longitudinal manner implied that the worsening effect among the CBPs might possibly be slower than those non-exposed over the 6-year period of study. A finding of an intra-individual change over time is statistically more efficient, and the methodological soundness to answer the research question was unfortunately not reported in the publication. A review (32) highlighted that a prospective study design would satisfy this major methodological challenge. In general, cross-sectional studies using effect biomarkers are limited for drawing causality inferences about the relationship between exposure to ENMs and observed effects, especially when the biomarkers reflect early biochemical or functional changes that lack clinical significance. Furthermore, co-exposures in the workplace and individual lifestyle factors require rigorous assessment to control for potential confounding biases. The authors concluded that only a longitudinal follow-up of a large number of workers exposed to (the same) ENMs could ultimately resolve the question of health risks (32).

### Consistency of the present review to other reviews of PSLT

According to the aforementioned criteria, the publications included in this review provide limited evidence of a conclusive relationship between inflammatory markers and biological responses (19, 33). Liou et al. (19) reviewed six published and nine unpublished epidemiological studies using biomarkers, among workers with exposure to ENMs, including nanosilver, carbon nanotubes, silica dioxide, TiO2, and nanoresin. CB was excluded from this review. The authors categorized the biological effects into lung, cardiovascular, immunological, and oxidative damages. Lung markers included increased lung fibrosis markers (serum TGF-ß1 and sputum KL-6); lung inflammatory markers, such as sputum IL-1ß and IL-8; fractional exhaled nitric oxide (FENO); and increased exhale breath condensate (EBC) biomarkers.

The findings were strongly dependent on the study design. All 11 cross-sectional studies reported positive relationships between the studied biomarkers and ENM exposures; three of four longitudinal studies showed a negative relationship; the fourth showed positive findings after a 1-year follow-up. The review by Liou et al. (17) enhanced the fact that the findings were strongly dependent on the study design; the cross-sectional studies found positive relationships between the biomarkers and health effects while the longitudinal studies failed to lead to consistent results.

Our review is in agreement with the conclusions of another review including two studies of CB (22, 24) that indicated limited evidence of adverse health effects with studied ENMs, based on the majority of cross-sectional studies reviewed (31).

## Summary

Based on the Chinese studies reviewed, evidence is lacking to “specific target organ toxicity” for respiratory systems under repeated exposure to CB. The five reviewed human studies of biological markers provide no clear or consistent evidence for adverse effects on lung function of clinical relevance or markers of airway inflammatory changes.

The inflammatory markers that were most studied failed to satisfy the evidentiary quality criteria. The significance of the results of the reviewed studies is limited by cross-sectional study design, inconsistency in results, unclear clinical relevance, and high occupational exposures. As a risk assessment relying on biological markers does not seem appropriate at this time. Nevertheless, the novel research warrants exploration and understanding of exposure to ENMs and potential health risks in an occupational setting. The major strength of the present review is the focus on the (same) exposure to CB rather than the evaluation of different ENMs.

The potential value and benefits of applying biomarkers for the risk assessment of ENMs are generally recognized. Valid data on the biomarkers, which require validation and standardization, are essential so that biomarkers can be used as decision-making tools in the future.

## Author contributions

MY: Conceptualization, Data curation, Formal analysis, Funding acquisition, Investigation, Methodology, Project administration, Resources, Software, Supervision, Validation, Visualization, Writing – original draft, Writing – review & editing. RM: Conceptualization, Funding acquisition, Investigation, Methodology, Project administration, Resources, Visualization, Writing – original draft, Writing – review & editing.

## References

[1] McCunney RJ Levy L Chaudhuri I Ngieweh Y Yong M Wampler W. Carbon black in Patty’s industrial hygiene and toxicology. eds. D. J. Paustenbach, W. Farland, H. Greim, J. Klaunig and L.Levy. 7th edn. New Jersey, USA: John Wiley & Sons, Inc. (2023).

[2] Creutzenberg O Pohlmann G Schaudien D Kock H. Toxicokinetics of nanoparticles deposited in lungs using occupational exposure scenarios. Front Public Health. (2022) 10:909247. doi: 10.3389/fpubh.2022.909247, PMID: 35801236 PMC9253415

[3] Warheit DB Kreiling R Levy LS. Relevance of the rat lung tumor response to particle overload for human risk assessment—update and interpretation of new data since ILSI 2000. Toxicology. (2016) 374:42–59. doi: 10.1016/j.tox.2016.11.013, PMID: 27876671

[4] ECETOC. Poorly Soluble Particles/Lung Overload-Technical Report 122, CEFIC-Brussels. Bruxelles: ECETOC (2023).

[5] Mauderly JL Snipes MB Barr EB Belinsky SA Bond JA Brooks AL. Pulmonary toxicity of inhaled diesel exhaust and carbon black in chronically exposed rats. Part I: Neoplastic and non- neoplastic lung lesions. Research Report No. 68. Cambridge, MA: Health Effects Institute (1994). 7530965

[6] Nikula KJ Snipes MB Barr EB Griffith WC Henderson RF Mauderly JL. Comparative pulmonary toxicities and carcinogenicities of chronically inhaled diesel exhaust and carbon black in F344 rats. Fundam Appl Toxicol. (1995) 25:80–94. doi: 10.1006/faat.1995.1042, PMID: 7541380 PMC7528960

[7] Heinrich U Fuhst R Rittinghausen S Creutzenberg O Bellmann B Koch W. Chronic inhalation exposure of Wistar rats, and two different strains of mice to diesel engine exhaust carbon black and titanium dioxide. Inhal Toxicol. (1995) 7:533–56. doi: 10.3109/08958379509015211

[8] Heinrich U. Inhalation of rats to tar/pitch condensation aerosol or carbon black alone or in combination with irritant gases In: Mohr U, editors. Toxic and carcinogenic effects of solid particles in the respiratory tract. Washington, DC: ILSI Press (1994)

[9] Yong M Anderle L Levy L McCunney RJ. Carbon black and lung Cancer mortality-a Meta-regression analysis based on three occupational cohort studies. J Occup Environ Med. (2019) 61:949–00. doi: 10.1097/JOM.0000000000001713, PMID: 31513042

[10] Morfeld P Mundt KA Dell LD Sorahan T McCunney RJ. Meta-analysis of cardiac mortality in three cohorts of carbon black production workers. Int J Environ Res Public Health. (2016) 13:302. doi: 10.3390/ijerph13030302 27005647 PMC4808965

[11] Bevan RJ Harrison PTC. Threshold and non-threshold chemical carcinogens: a survey of the present regulatory landscape. Regul Toxicol Pharmacol. (2017) 88:291–02. doi: 10.1016/j.yrtph.2017.01.003 28119000

[12] Carter JM Corson N Driscoll KE Elder A Finkelstein JN Harkema JN. Comparative dose-related response of several key pro- and anti- inflammatory mediators in the lungs of rats, mice, and hamsters after subchronic inhalation of carbon black. J Occup Environ Med. (2006) 48:1265–78. doi: 10.1097/01.jom.0000230489.06025.14, PMID: 17159643

[13] Brook RD. Cardiovascular effects of air pollution. Clin Sci (Lond). (2008) 115:175–87. doi: 10.1042/CS20070444 18691154

[14] Brook RD Rajagopalan S Pope CA III Brook JR Bhatnagar A Diez-Roux AV. Particulate matter air pollution and cardiovascular disease: an update to the scientific statement from the American Heart Association. Circulation. (2010) 121:2331–78. doi: 10.1161/CIR.0b013e3181dbece1 20458016

[15] Newby DE Mannucci PM Tell GS Baccarelli AA Brook RD Donaldson K. Expert position paper on air pollution and cardiovascular disease. Eur Heart J. (2015) 36:83–93. doi: 10.1093/eurheartj/ehu458 25492627 PMC6279152

[16] Menetski JP Hoffmann SC Cush SS Kamphaus TN Austin CP Herrling PL. The Foundation for the National Institutes of Health Biomarkers Consortium: past accomplishments and new strategic direction. Clin Pharmacol Ther. (2019) 105:829–843. doi: 10.1002/cpt.1362 PMC6593617 30648736

[17] Temple RJ. A regulatory authority’s opinion about surrogate endpoints In: Nimmo WS Tucker GT, editors. Clinical measurement in drug evaluation. New York: John Wiley and Sons Inc. (1995)

[18] Temple R. Are surrogate markers adequate to assess cardiovascular disease drugs? JAMA. (1999) 282:790–5. doi: 10.1001/jama.282.8.790 10463719

[19] Liou SH Tsai CS Pelclova D Schubauer-Berigan MK Schulte PA. Assessing the first wave of epidemiological studies of nanomaterial workers. J Nanopart Res. (2015) 17:413–38. doi: 10.1007/s11051-015-3219-7, PMID: 26635494 PMC4666542

[20] Page MJ McKenzie JE Bossuyt PM Boutron I Hoffmann TC Mulrow CD. The PRISMA 2020 statement: an updated guideline for reporting systematic reviews. BMJ. (2021) 372:n71. doi: 10.1136/bmj.n71 33782057 PMC8005924

[21] Leptak C Menetski JP Wagner JA Aubrecht J Brady L Brumfield M. What evidence do we need for biomarker qualification? Sci Transl Med. (2017) 9:eaal4599. doi: 10.1126/scitranslmed.aal4599, PMID: 29167393

[22] Cao X Lin L Sood A Ma Q Zhang X Liu Y. Small airway wall thickening assessed by computerized tomography is associated with low lung function in Chinese carbon black packers. Toxicol Sci. (2020) 178:26–35. doi: 10.1093/toxsci/kfaa134, PMID: 32818265 PMC7825005

[23] Yang M Li Y Meng T Zhang L Niu Y Dai Y. Ultrafine CB-induced small airway obstruction in CB-exposed workers and mice. Sci Total Environ. (2019) 671:866–73. doi: 10.1016/j.scitotenv.2019.03.033, PMID: 30947057

[24] Zhang R Dai Y Zhang X Niu Y Meng T Li Y. Reduced pulmonary function and increased pro-inflammatory cytokines in nanoscale carbon black-exposed workers. Part Fibre Toxicol. (2014) 14:73. 10.1186/s12989-014-0073-1 PMC4318129 25497989

[25] Cheng W Liu Y Tang J Duan H Wei X Zhang X. Carbon content in airway macrophages and genomic instability in Chinese carbon black packers. Arch Toxicol. (2020) 94:761–71. doi: 10.1007/s00204-020-02678-6, PMID: 32076763

[26] Dai Y Niu Y Duan H Bassig BA Ye M Zhang X. Effects of occupational exposure to carbon black on peripheral white blood cell counts and lymphocyte subsets. Environ Mol Mutagen. (2016) 57:615–22. doi: 10.1002/em.22036, PMID: 27671983 PMC6759205

[27] Poland CA Duffin R Weber K Dekant W Borm PJA. Is pulmonary inflammation a valid predictor of particle induced lung pathology? The case of amorphous and crystalline silicas. Toxicol Lett. (2023) 14:S0378–4274. doi: 10.1016/j.toxlet.2023.07.012 37454774

[28] Valent P Klion AD Horny HP Roufosse F Gotlib J Weller PF. Contemporary consensus proposal on criteria and classification of eosinophilic disorders and related syndromes. J Allergy Clin Immunol. (2012) 130:607–612.e9. doi: 10.1016/j.jaci.2012.02.019, PMID: 22460074 PMC4091810

[29] Pearson K. Notes on the history of correlations. Biometrika. (1920) 13:25–45. doi: 10.1093/biomet/13.1.25

[30] Altman DG Altman E. Practical statistics for medical research. Boca Raton: Chapman & Hall/CRC (1999).

[31] Judd CM Kenny DA. Data analysis In: Gilbert D Fiske ST Lindzey G, editors. The handbook of social psychology. 5th ed. New York: John Wiley & Sons, Inc. (2010)

[32] Guseva Canu I Schulte PA Riediker M Fatkhutdinova L Bergamaschi E. Methodological, political and legal issues in the assessment of the effects of nanotechnology on human health. J Epidemiol Community Health. (2018) 72:148–53. doi: 10.1136/jech-2016-208668, PMID: 29203525 PMC6262826

[33] Schulte PA Leso V Niang M Iavicoli I. Current state of knowledge on the health effects of engineered nanomaterials in workers: a systematic review of human studies and epidemiological investigations. Scand J Work Environ Health. (2019) 45:217–38. doi: 10.5271/sjweh.3800 30653633 PMC6494687

[34] FDA-NIH Biomarker Working Group. BEST (biomarkers, EndpointS, and other tools) Resource (2016). Available at: https://www.ncbi.nlm.nih.gov/books/NBK326791/27010052

